# Practice environment determinants of job satisfaction among midwives at healthcare facilities in Accra Metropolis: A multicentre study

**DOI:** 10.1371/journal.pone.0282251

**Published:** 2023-03-01

**Authors:** Dorothy Akua Aikins, Collins Atta Poku, Ernestina Donkor, Florence Naab

**Affiliations:** 1 Nursing and Midwifery Training College, Accra, Ghana; 2 Department of Nursing, Kwame Nkrumah University of Science and Technology, Kumasi, Ghana; 3 School of Nursing and Midwifery, University of Health and Allied Science, Ho, Ghana; 4 School of Nursing and Midwifery, University of Ghana, Accra, Ghana; National Defence University - Kenya, KENYA

## Abstract

**Introduction:**

Sustainable Development Goal 3 (SDG 3) has been one of the key goals for all partners of health globally. The health workforce especially midwives are among the principal skilled experts for achieving the goal. This is evidenced in the role they play in caring for pregnant women from the antenatal stages to the postpartum periods. However, very little has been reported about midwives’ job satisfaction in Ghana. The study assessed the practice environment determinants of job satisfaction among registered midwives in Ghana.

**Materials and methods:**

A cross-sectional design was adopted to recruit midwives from public and quasi-government hospitals in Accra Metropolis. Validated scales—‘Measure of Job Satisfaction’ and ‘Practice Environment Scale of Nursing Work Index’ were used for data collection. Data was analysed through descriptive statistics, Pearson correlation and linear regression.

**Results:**

Midwives had a positive perception of their work environment. They were generally satisfied with their job but were dissatisfied with their salaries. Key determinants of midwives’ job satisfaction included years of work as a midwife, managers’ leadership and support, and adequacy of human and material resources.

**Conclusion:**

Improving midwives’ job satisfaction through enabling the work environment will go a long way to improve healthcare provision in the healthcare delivery points in helping achieve SDG 3.

## Introduction

Sustainable Development Goal 3 (SDG 3) has been one of the key goals for all partners of health globally. SDG 3 is targeted at reducing to a level of less than 70 per 100,000 live births of global maternal mortality rate, decreasing neonatal mortality to at least 12 per 1,000 live births and under-5 mortality to at least 25 per 1,000 live births by stopping preventable deaths of mothers, newborns and children under the age of five by 2030 [1, 2].

An organisation with a strong and sustainable workforce is likely to achieve quality healthcare services. A strong and healthy workforce is a great tool for effective health service delivery and achieving excellent health outcomes. The health workforce especially midwives are among the principal experts for achieving Goal 3 of the SDG [3, 4]. As a result, health organisations consider human resources as a vital asset in the healthcare systems without which knowledge cannot be imparted and care cannot also be affected. In the area of service delivery, employee behaviour can either tarnish the image or bring success and quality service to an organisation. Therefore, employees are viewed as the most important asset to enhance the creation of wealth if only they are pleased with their job and remain faithful to them [5, 6].

It is estimated that a working individual spends at least six hours of their time at work and therefore must get some satisfaction from it which will, in turn, impact productivity. Since one’s job and the work environment become part of the individual, it behoves both the employer and the employee to assess their satisfaction level for better job outcomes. This is because a satisfied workforce will put in additional effort to meet organizational goals [7, 8].

Job satisfaction in an organisation is regarded as an important measure of high productivity and improved work. There is a relationship between staff turnover, non-attendance of employees, workplace accidents and job satisfaction. Job satisfaction is an extremely vital phenomenon of study because globally, it has been established to determine quality care and patient safety [9]. According to Locke (p. 317), job satisfaction is defined as a “pleasurable emotional state of the appraisal of one’s job, as achieving or facilitating one’s job value” [10]. In effect, one who likes his or her work may be considered as having job satisfaction and vice versa.

A Global shortage of healthcare workers, including nurses and midwives, has been a worrying phenomenon over the years. According to World Health Statistics on the health workforce, it is predicted that the world would have a shortage of over 14 million healthcare workers by 2030 if the current development does not change. Currently, there is an expected shortage of about over 9 million nurses and midwives; and the regions that have been predicted to be highly affected are Sub-Saharan Africa (SSA) and South-East Asia [11, 12]. The health workforce shortage in SSA is quite disturbing. In the past decade, the average doctor-to-patient ratio in SSA was recorded to be 2 doctors per 10,000 population and 11 nurses or midwives per 10,000 population, compared with 19 doctors and 49 nurses or midwives per 10,000 for the Americas, and 32 doctors and 78 nurses or midwives per 10,000 for Europe [13]. Anecdotal evidence suggests that the situation has improved; it is still estimated that by 2035, the world would have recorded a 12.7 million shortage of health workforce [14]. Moreover, the global midwifery report showed that the number of midwifery staff to provide healthcare to women and new-borns is woefully inadequate with 73 countries in the world confronted with critical deficiencies of midwives, and this can lead to preventable mortality of mothers and/ or the new-borns [15–17].

The shortage of the nursing workforce is the most impactful in Africa [18], as they form a chunk of the health workforce. This episode contributes to defeating the aims of healthcare systems worldwide making it difficult for nurses/midwives to meet the health needs of their people [19].

In Ghana, nurses and midwives work in very challenging work environments which include a shortage of staff, work overload, inadequate logistics coupled with the poor interpersonal relationship between midwives and labouring mothers. In addition to this, inadequate training capacity, ineffective management structures in place and poor working conditions coupled with limited financial and non-financial motivation contribute to increased attrition and poor morale and performance [20–22]. Yet they have been identified as the bigger workforce to help sustain the SDG 3 Target 1; Global maternal mortality rate (MMR) should be below 70 per 100,000 live births by 2030 [2].

The Greater Accra Region of Ghana is equally faced with problems of reproductive health services and high MMR. Accra Metro Health Directorate reports indicated that maternal death has remained high despite all attempts to reduce it. In the year 2019, the metropolis recorded an institutional maternal mortality rate (MMR) of 305 per 100,000 live births. There was a decrease in MMR in 2020, which recorded 277 per 100,000 live births and in 2021 had further increased to 359 per 100,000 live births as stated in the Greater Accra Region Human Resource Directorate 2019 Report [23]. Despite all the challenges, Ghanaian midwives continue to work hard to save mothers and their babies but there is a paucity of data on midwives’ job satisfaction in SSA, especially in Ghana. The study assessed the relationship between job satisfaction and practice environment and the determinants of midwives’ job satisfaction in the Accra Metropolis.

## Materials and methods

### Research design and setting

A cross-sectional research design was employed in this research work. The study setting was the Accra metropolis. The metropolis is situated in the southeastern part of Ghana along the Gulf of Guinea. Seven public and three quasi-government hospitals were randomly selected namely: Achimota Hospital, Greater Accra Regional Hospital, Adabraka Polyclinic, Ussher Polyclinic, Maamobi General Hospital, Kaneshie Polyclinic, Mamprobi Polyclinic, Police Hospital, the Trust Hospital and the University of Ghana Hospital. Greater Accra numbers 970 of which 321 (30.2%) were in Accra Metropolis [23].

### Study population

The target participants included all midwives working in the selected facilities. Inclusions were all midwives who had worked for not less than one year in antenatal clinics, labour wards, postnatal wards, obstetrics and gynaecology wards, family planning units, obstetric emergency units, obstetric theatres and recovery wards or obstetric out-patient departments in the facility. Midwives with administrative roles and those on leave were excluded from the study.

### Sampling and sample size

Slovin’s formula was used to estimate a sample size of 196 midwives. A multistage sampling approach was used as the sampling technique; where a simple random technique was adopted to select 10 health facilities from a known sample frame of fifteen public health facilities and five quasi-government hospitals. A proportionate stratified sampling was used to sample the 196 participants from the public health (148 midwives) and quasi-government (48 midwives) facilities through a convenient sampling technique.

### Measures

Validated scales Practice Environment Scale of Nursing Work Index (PES-NWI) and Measure of Job Satisfaction (MJS) were used for data collection.

#### Practice Environment Scale of Nursing Work Index (PES-NWI)

Midwives’ practice environment was measured using PES-NWI developed by Lake [24]. The scale is made up of thirty-two items under five subscales: Nurse Manager Leadership, Ability and Support (11 items); Collegial Midwife-Physicians Relation (7-items); Staffing and Resource Adequacy (7-items); Midwives Participation in Hospital Affairs (3-items), and Nursing Foundation for Quality Care (7-items). It is measured on a 4 Likert scale ranging from 0–3 with strongly disagree (0) to strongly agree (3). PES-NWI has been used in other settings with acceptable Cronbach alpha coefficients between 0.64–0.91 [25, 26].

#### Measure of Job Satisfaction (MJS)

The MJS questionnaire by Traynor and Wade [27] was used to measure midwives’ Job satisfaction. The scale has 6 subscales including personal satisfaction (6 items), satisfaction with workload (7 items), satisfaction with salary (4 items), satisfaction with professional support (13 items), satisfaction with prospects (6 items) and satisfaction with standards (6 items). The scale is a 5-point Likert scale from ‘strongly dissatisfied’ (1) to ‘strongly satisfied’ (5). MJS has reported an acceptable Cronbach alpha coefficient of 0.71 to 0.94 in other studies [28, 29].

### Data collection procedure

The participants for the study were conveniently selected and informed about the purpose of the study. Those who agreed to be part of the study were recruited for the study. Self- administered questionnaires were administered to participants and completed questionnaires were collected immediately after they were answered.

### Data analysis

IBM SPSS (version 25) was used to analyse the data. Data cleaning was done by computing the frequencies for all the variables to confirm the accuracy of the data entered. Descriptive statistics like frequencies, means and standard deviations were used. Correlation analysis was used to establish a relationship between socio-demographic data, practice environment and job satisfaction of the midwives. Also, to determine the predictors of job satisfaction, a multiple linear regression analysis was conducted at a significance of *p<0*.*05* after all the statistical assumptions were tested.

### Ethical consideration

Ethical clearance was obtained from the IRB of the NMIMR (CPN 043/16-17) while permission to use the selected facilities was also sought from the Greater Accra Regional Health Directorate. A thorough explanation of the study was given to the participants to obtain their consent and each individual consented to participate in the study. Participants were also told that they had a right to withdraw from the research at any point in time. Anonymity and confidentiality were also ensured as participants did not write their names on the questionnaire.

## Results

### Socio-demographic characteristics

As presented in Table 1, from an eligible 196 participants, 183 questionnaires were retrieved, giving a response rate of 93.4%. All participants were females and most of them (73.2%, n = 134) were married. The majority of the participants (51.4%; n = 94) were between the ages of 26 and to 35years. In terms of academic qualifications, the majority of the participants (49.2%, n = 90) were diploma midwifery holders, and staff midwives formed the majority (35%, n = 64) in terms of rank. Almost forty per cent (37.2%, n = 68) of the participants reported they have worked as midwives for a period between 1–3 years, and close to half of the participants (44.3%, n = 81) indicated that they work at the obstetric/maternity unit.

**Table 1:** Demographic characteristics of participants.

Variable		Frequency (n)	Per cent (%)
Age groups of participants	18–25	6	3.3
	26–35	94	51.4
	36–45	31	16.9
	46–55	31	16.9
	Above 55	21	11.5
Marital status of participants	Single	41	22.4
	Married	132	72.1
	Separated	4	2.2
	Divorced	2	1.1
	Widowed	4	2.2
Qualification	Certificate	74	40.4
	Diploma	90	49.2
	Bachelors	16	8.8
	Master of nursing/midwifery	3	1.6
Years of work as a Registered Midwife	1-3years	68	37.2
	4-6years	42	23.0
	7-9years	22	12.0
	10years and above	51	27.9
Unit of work	OPD/Obstetric emergency	11	6.0
	Antenatal/Postnatal wards	53	29.0
	Maternity/Obstetric wards	81	44.3
	Obstetric/Gynae ward	22	12.0
	NICU	1	.5
	Obstetric theatre	1	.5
	Family planning	5	2.7
	Others	9	4.9

### Job satisfaction of midwives

The levels of job satisfaction among the midwives were assessed as presented in Table 2. The composite mean score of overall job satisfaction was 3.05 (SD = 0.56). The composite mean scores of the various sub-scales are as follows: personal satisfaction on the job (n = 3.60), satisfaction with workload (n = 3.21), satisfaction with professional support (n = 3.01), satisfaction with prospects (n = 3.82) and satisfaction with the standard of care (n = 2.67). However, satisfaction with their pay had a low composite mean score of 2.01 (SD = 0.33).

**Table 2:** Job satisfaction of midwives.

Variables	Composite Mean	SD
Personal satisfaction	3.60	0.59
Satisfaction with workload	3.21	0.70
Satisfaction with professional support	3.01	0.63
Satisfaction with pay	2.01	0.33
Satisfaction with prospect	3.82	0.67
Satisfaction with standards of care	2.67	0.45
Overall satisfaction	3.05	0.56

### Relationship between professional practice environment and job satisfaction

The Pearson Product Moment Correlation was used to examine the relationship between midwives’ practice environment and overall job satisfaction. The results are presented in Table 3. The results showed midwives’ job satisfaction to have a moderately significant correlation with all the facets of the practice environment; nurse manager leadership, ability and support (r = .444, p < .001), collegial nurse-physician relationship (r = .349, p < .001), staffing and resource adequacy (r = .356, p < .001), the nursing foundation for quality of care (r = .406, p < .001). However, midwives’ participation in hospital affairs showed a weak but statistically significant positive relationship with job satisfaction (r = 142, p < .006). Thus, an increase in any of the parameters of the work environment leads to a corresponding increase in midwives’ job satisfaction.

**Table 3:** Correlations between the practice environment dimension and overall job satisfaction.

**Variables**	**1**	**2**	**3**	**4**	**5**
1. Nurse Manager Leadership, Ability and Support	-				
2. Collegial Nurse–Physician Relations	.622**	-			
3. Staffing and Resource Adequacy	.752**	.702**	-		
4. Midwives’ Participation in Hospital Affairs	.504**	.550**	.260	-	
5. Nursing Foundation for Quality of Care	.494**	.327**	.368	.188*	-
6. Overall job satisfaction	.444**	.349**	.356**	.142*	.406**

** = Correlation is significant at 0.01 level (2-tailed),

* = Correlation is significant at 0.05 level (2-tailed)

### Predictors of midwives’ job satisfaction

A multiple linear regression analysis was used to determine the predictors of midwives’ job satisfaction in the model as presented in Table 4. In the model, the five components of midwives’ work environment significantly explained 48.9% of the variance in job satisfaction [R^2^ = .489, F_(8, 174)_ = 20.784, p = .001]. Nurse manager leadership, ability and support, collegial midwife-physician relations, staffing and resource adequacy contributed 29%, 7.4% and 22.3% respectively to the model. Additionally, midwives’ participation in hospital affairs and nursing foundation for quality care contributed 10.0% and 15.1% respectively. In examining the variables, four of them emerged as significant predictors of job satisfaction in the model: nurse manager leadership, ability and support (p < .001), staffing and resource adequacy (p < .001) and nursing foundation for quality of care (p < .05).

**Table 4:** Predictors of midwives’ job satisfaction.

Variables	B	SE	Beta	T	Sig.	R
(Constant)	31.346	20.660		1.517	.131	
Nurse Manager, Leadership, Ability and Support	1.341	.270	.290	4.962	.000	.444
Collegial Nurse-Physician Relations	.544	.451	.074	1.206	.230	.349
Staffing and Resource Adequacy	1.443	.377	.223	3.824	.000	.356
Midwives’ Participation in Hospital Affairs	2.029	1.108	.100	1.830	.069	.142
Nursing Foundation of Quality of Care	2.494	.998	.151	2.500	.013	.406
Summary: R^2^ = .489, F_(5, 177)_ = 20.784, p < .001						

Dependent variable: Job satisfaction 95% confidence level (α = .05)

## Discussion

The roles of organisations are numerous but one most important roles are to satisfy their workers because a satisfied employee works efficiently and effectively to achieve organisational goals [30, 31]. The findings of the current study suggested that midwives were satisfied with their work which is congruent with the findings of other studies [32–34]. Similarly, Munyewende et al. [35] in South Africa reported that nurses’ job satisfaction was relatively high. Though midwives indicated their satisfaction with the workload, the reason could be attributed to the high number of professional midwives in the Accra metropolis. However, the findings of the current study on the salaries of midwives revealed that they were not satisfied considering their vital role in achieving SDG3 and the increased workload due to their limited numbers in healthcare delivery. This finding is consistent with other studies that reported that nurses in the Philippines, Nigerian and Ethiopian nurses were moderately satisfied with their job but dissatisfied with their salaries [36–38]. Other studies with similar findings include Öncü et al [39], Okafor and Chimereze [40], Hashish and Ashour [41], and Semachew et al [42], in addition, reported that poor salary was one of the factors that pushed health workers to migrate to the Western world. Therefore, poor salary aside from other findings may determine the midwives’ job satisfaction.

With the moderate correlation between job satisfaction and facets of the work environment, previous research work by Klopper et al [43], Cummings et al [44] and Dhamija, Gupta and Bag [45] reported similar findings. Apart from improving the midwifery workforce, there is a need for authorities to also include young midwives in decision-making because they form the bulk of the working population.

In the current study, job satisfaction among midwives’ was predicted by the number of years RM have worked, nurse manager leadership, ability and support, staffing and resource adequacy, and nursing foundation for quality of care. Previous studies by Aloisio et al [46], Lu et al [47], and Hayes et al [34] reported that years nurses have worked have an impact on stress and burnout which eventually affects job satisfaction. The implication is that when people gain experience over the years, it helps them to manage their stress better. Likewise, Mousazadeh et al [48] explain that older nurses had job satisfaction, a higher probability of promotion and good life. Again, poor management practices and resource inadequacy are important indicators of midwives’ dissatisfaction with their jobs [49]. Human and material resource inadequacy continues to pose a challenge to improving maternal health and the SDG 3 in SSA including Ghana. Midwives are the key service providers of care and support for mothers throughout pregnancy, delivery and puerperium, and newborns at the critical stage in their lives [38]. Every effort must be made to keep midwives happy at the post so that they will work hard toward reducing maternal mortality in the facilities. Though Skilled Birth Attendants (SBA) are helping with the shortfall, the disproportion of high numbers of SBA as against RMs is an area of concern in the sub-region. Even though there has been an improvement in the number of midwives trained and SBA in recent times in Ghana, there must be an equitable regional distribution of SBA [50] to curtail increased workload to improve job satisfaction.

## Conclusion

The practice environment is a significant determinant of nurses’ job satisfaction, according to recent research. Additionally, these characteristics interact with one another, indicating that managerial activities intended to increase workplace satisfaction should consider both aspects of the leadership role and employment setting. Nurse managers need to be careful to keep the work environment and people’s attitudes in sync with one another. Job satisfaction can be increased by improving the workplace, but this effect can be strengthened by prioritizing strong material and people resources and by upholding intrinsic rather than external work values. Higher job satisfaction for nurses can result from working in a setting that offers consistent and coordinated good working conditions, competitive compensation, and a solid infrastructure for providing high-quality nursing care. This also seems to suggest that using compensation alone won’t be sufficient to increase midwives’ job happiness. This feature may also aid in our understanding of the well-known conundrum that increased pay does not necessarily translate into higher job satisfaction in the context of healthcare, but another factor including nurse managers’ leadership and support.

## Abbreviations

IRB: Institutional Review Board
MJS: Measure of Job Satisfaction
MMR: Maternal Mortality Rate
NICU: Neonatal Intensive Care unit
NMIMR: Noguchi Memorial Institute for Medical Research
OPD: Out-patient Department
PES-NWI: Practice Environment Scale of Nursing Work Index
SBA: Skilled Birth Attendant
SDGs: Sustainable Development Goals
SSA: Sub-Saharan Africa
